# Twisting phase and intensity of light with plasmonic metasurfaces

**DOI:** 10.1038/s41598-018-23382-7

**Published:** 2018-03-20

**Authors:** Yuchao Zhang, Xiaodong Yang, Jie Gao

**Affiliations:** Department of Mechanical and Aerospace Engineering, Missouri University of Science and Technology, Rolla, MO 65409 USA

## Abstract

Twisting light in both phase and intensity has recently drawn great interests in various fields related to light-matter interactions such as optical manipulation of particles and quantum entanglement of photons. Conventionally, bulky optical components are required to produce such twisted optical beams, which significantly limits their applications in integrated photonics and optical chips. Here, we design and demonstrate aluminum plasmonic metasurfaces consisting of nanoslit antennas as ultracompact beam converters to generate the focused twisted beams in both phase and intensity across the visible wavelength range. The metasurface is encoded with the combined phase profile containing the helico-conical phase function together with a Fourier transform lens based on the Pancharatnam-Berry (PB) geometric phase. It is demonstrated that the created twisted beams simultaneously possess three-dimensional (3D) spiral intensity distribution around the propagation axis and complex phase structure containing both the central vortex and the peripheral vortex string. Moreover, the twisted beam exhibits an arithmetic intensity spiral at the focal plane with the maximum photon concentration located at the leading point of the spiral. Our results show the promising potential for advancing metasurface-based integrated devices in many applications of light-matter interactions.

## Introduction

Optical vortices having helical phase profiles and phase singularities have drawn considerable attention in many exciting areas related to light-matter interactions, such as quantum optics^1–5^, high-resolution imaging^6,7^, optical communications^8,9^, and optical manipulation of particles^10–12^. The phase profile of optical vortex is expressed by $\exp (il\phi )$, where $\phi$ is the azimuthal angle and *l* represents the topological charge (TC). The intensity distribution of optical vortex exhibits the doughnut-shaped ring with the ring radius depending on the TC. As the TC is increased, the vortex ring radius becomes larger and the photon density decreases, which has limitations for applications requiring both large orbital angular momentum (OAM) and high photon concentration, such as cold atom rotation^13–15^ and quantum entanglement of photons^16,17^. One solution for the above issue is to consider optical beams with both twisted phase and intensity profiles^18^ so that TC-independent high photon density can be realized in the focused areas. One important twisted beam in both phase and intensity is helico-conical (HC) beam^18–22^, which can be used to introduce spiral motion on the trapped particles by the transfer of OAM. The conventional method to generate HC beam requires free-space bulky optical components including spatial light modulator and Fourier transform lens, which increases the optical system complexity and also limits the photonic chip integration.

In recent years, plasmonic metasurfaces made of nanoantenna arrays in ultrathin metallic films have provided a powerful and functional platform for tailoring the phase, intensity and polarization of light at the subwavelength scale^23,24^. By introducing the Pancharatnam-Berry geometric phase accompanied with polarization conversion^25–31^, metasurfaces have been widely used for building on-chip wavefront shaping devices such as optical vortex generators^32–36^, flat optical lenses^37–42^ compact wave plates^43–45^, and multiplexed holograms^46–51^. Although noble metals like gold and silver have been widely employed in plasmonic metasurfaces in the visible spectrum, gold has limitations for working below the wavelength of 550 nm due to the interband transition and silver is susceptible to oxidation and sulphidation under ambient condition. Alternatively, aluminum has been identified as a promising substitute for gold and silver for plasmonic metasurfaces operating within the visible and near-UV frequency regions^47,52–55^ due to its high plasma frequency, chemical and thermal stability thanks to its natively formed oxidation layer, low cost, and complementary metal oxide semiconductor (CMOS) compatibility.

In this work, we present aluminum plasmonic metasurfaces constructed from nanoslit antenna arrays to generate HC beams with both twisted phase and intensity profiles in the visible wavelength range from 400 nm to 800 nm. The metasurface with compact area of 50 μm by 50 μm is encoded with the PB phase profile created by combining the HC phase function and the phase of Fourier transform lens. It is shown that the produced HC beams display 3D spiral intensity trajectory around the propagation axis and complex phase structure containing both the central vortex and the peripheral vortex string. We also demonstrate that the HC beam with varying TC always exhibits an arithmetic intensity spiral at the focal plane where the maximum photon density is concentrated at the leading point of the spiral. Our demonstrated results will provide new opportunities for realizing metasurface-based photonic devices used in various applications of light-matter interactions such as quantum information processing, optical trapping, and optical communications.

## Results

### Design of aluminum metasurface for HC beam generation

**Figure 1:**
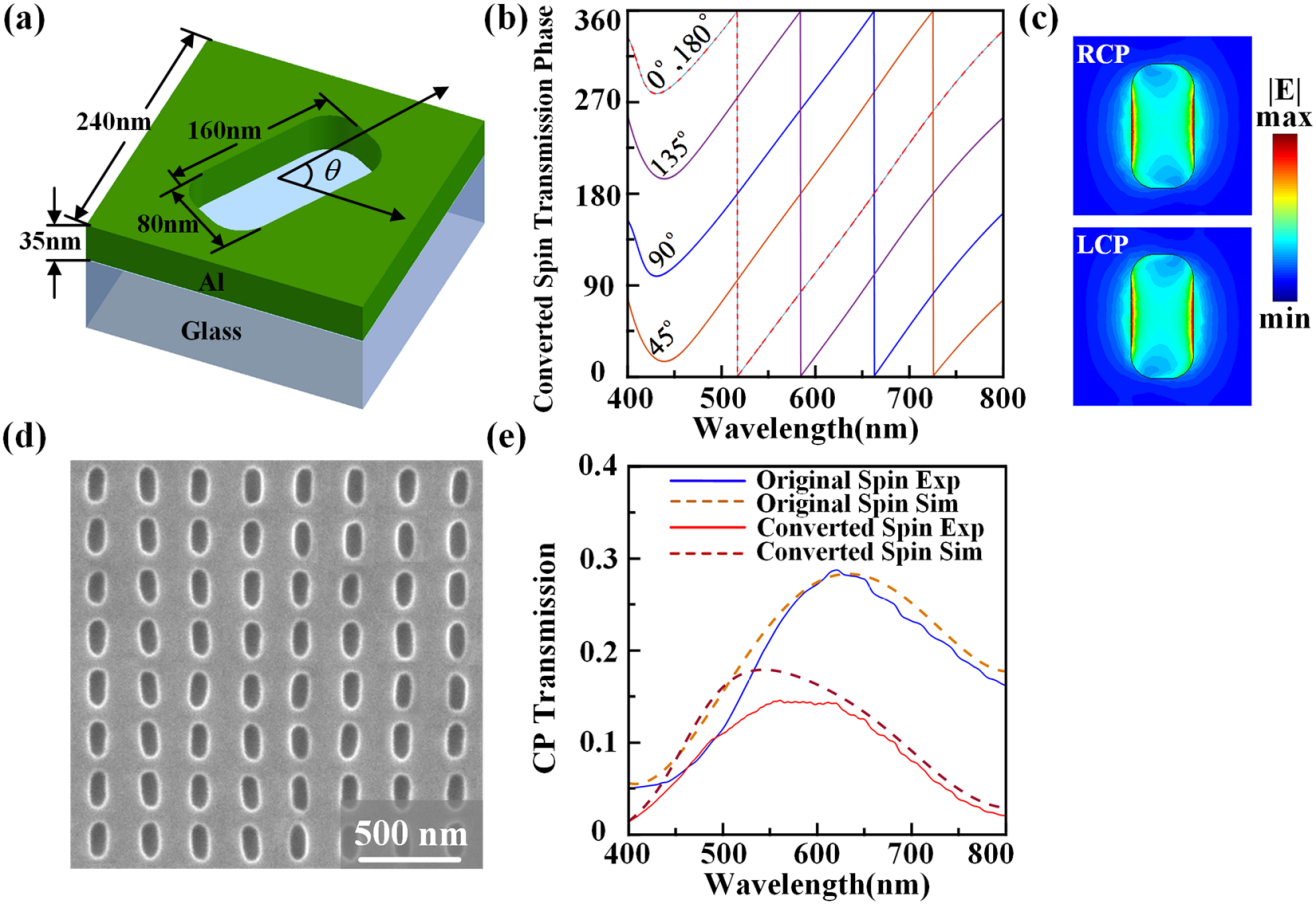
(**a**) Schematic of the unit cell design of the nanoslit antenna at rotation angle of *θ*. (**b**) Simulated geometric phase shifts for the transmitted converted spin component from the nanoslit antennas at different rotation angles of 0, 45°, 90°, 135°, and 180°. (**c**) Simulated electric field |*E*| distributions of nanoslit antenna under circular polarizations at 532 nm. (**d**) A SEM image of the fabricated homogeneous nanoslit antenna array. (**e**) Measured and simulated transmission spectra under circular polarization basis.

The metasurface with compact size of 50 μm by 50 μm contains 208 by 208 subwavelength nanoslit antennas with different orientation angles. As shown in Fig. 1(a), the nanoslit antennas are etched in a thin aluminum film with thickness of 35 nm on glass substrate using focused ion beam (FIB) method. The width and length of each nanoslit antenna is 80 nm and 160 nm respectively, and the unit cell period is 240 nm. The introduced PB phase from nanoslit antenna is only determined by the orientation angle *θ* of the nanoslit. When the incident circularly polarized beam normally transmits through the anisotropic nanoslit antenna, the transmitted beam will contain both the original spin component with no phase shift and the converted spin component with the induced PB phase shift of 2*θ* which is twice as the nanoslit rotation angle. The overall phase profile encoded in the metasurface is obtained by arranging the nanoslit antennas with designed rotation angles to form spatially inhomogeneous array. Figure 1(b) plots the simulated geometric phase shifts for the transmitted converted spin component from the nanoslit antennas at different rotation angles, showing that the phase shift variations of the converted spin component from the nanoslit antennas are twice as their rotation angles in the broad wavelength range from 400 to 800 nm. The simulated electric field |*E*| distributions of the nanoslit antenna under circular polarization basis (left-handed and right-handed circular polarizations, LCP and RCP) at 532 nm are shown in Fig. 1(c), indicating strong polarization anisotropy of the nanoslit. The SEM image of the fabricated homogeneous array of nanoslit antenna is shown in Fig. 1(d). Next, the transmission spectra under circular polarization basis are measured and simulated in Fig. 1(e), where the original spin component has LCP (blue solid line) and the converted spin component has RCP (red solid line). It is observed that the converted spin component reaches to the maximum transmission of around 15% near the wavelength of 550 nm, where the plasmonic resonance occurs. The simulation results (dot lines) match well with the experimental data, proving the effectiveness of the designed nanoslit antennas.

**Figure 2:**
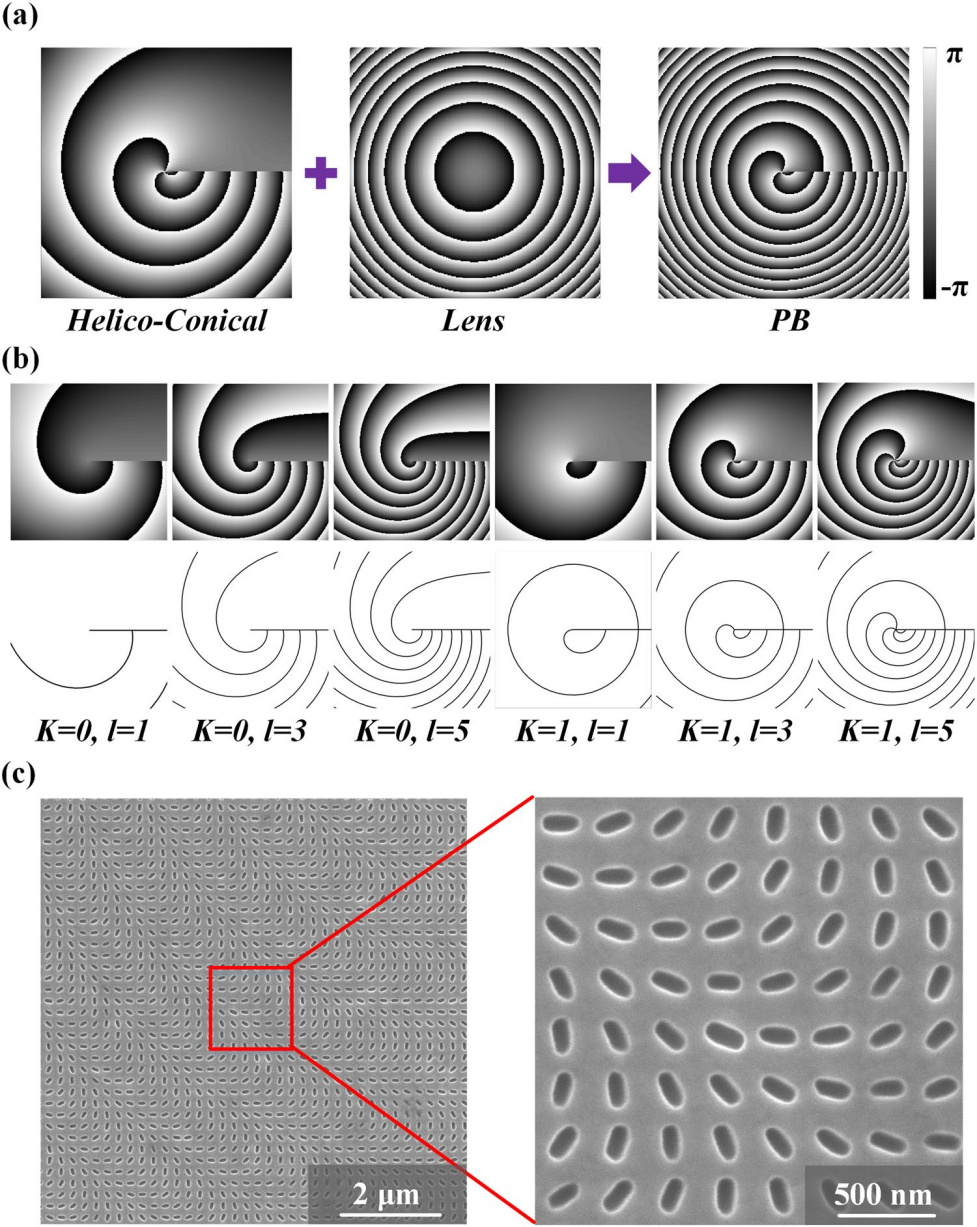
(**a**) Phase profile of metasurface to generate HC beam with *K* = 1 and *l* = 3. (**b**) The HC phase functions with different parameters (upper row) and the corresponding phase contour lines (lower row). (**c**) SEM images of the fabricated metasurface sample.

The metasurface is formed by rotating the nanoslit antenna within each unit cell with the orientation angle *θ*(*x*, *y*) determined by half of the transmitted PB phase shift *φ*_*PB*_(*x*, *y*) of the converted spin component. The generated PB phase profile *φ*_*PB*_(*x*, *y*) is a superposition of the phase distributions of HC phase and Fourier transform lens, as shown in Fig. 2(a). The PB phase profile is defined by the following functions,

1 $${\phi }_{PB}(x,y)={\phi }_{HC}(x,y)+{\phi }_{lens}(x,y)$$

2 $${\phi }_{HC}(x,y)=l\cdot \text{arg}(x+yi)\cdot (K-\frac{\sqrt{{x}^{2}+{y}^{2}}}{{r}_{0}})$$

3 $${\phi }_{lens}(x,y)=\frac{\pi }{\lambda }[\sqrt{({x}^{2}+{y}^{2})+{f}^{2}}-f]$$

where *x*, *y* represent the position coordinates of the unit cell in the specific column and row in the antenna array with respect to the center of the array. In the polar coordinates, Eq. (2) is expressed as *φ*_*HC*_(*r*, *θ*) = *lθ*(*K* − *r/r*_0_), which defines a HC phase function with the product of a helical phase and a conical phase. The parameters *l* is the TC of the HC beam, *K* takes the value of either 0 or 1, and *r*_0_ determines the period of the conical phase. Compare to common Laguerre–Gaussian optical vortex beams, the HC beam contains nonseparable radial and azimuthal terms, and the generated far-field intensity has spiral structure. Eq. (3) introduces the phase profile of spherical wave for a Fourier transform lens with the focal length of $f$, and *λ* is the operation wavelength.

The PB phase distribution shown in Eq. (1) will directly create a focused HC beam. In our design, the focal length of the Fourier transform lens is chosen to be *f* = 200 μm at the wavelength of 532 nm. The HC phase function contains three parameters including TC parameter *l* = 1, 3 and 5, the parameter *K* = 0 or 1, and the conical phase parameter *r*_0_ = 5 μm. Therefore, six metasurface samples are fabricated and characterized for generating HC beams with the above selected parameters. In Fig. 2(b), the phase distributions (upper row images) and phase contours (lower row images) of the six different HC phase functions are plotted, where the phase contour lines are located at *φ*_*HC*_ = 0. It is observed that the HC phase profiles are featured with spiral structures and horizontal discontinuous cut lines. As the TC increases, the interval between two adjacent contour lines become smaller, hence the phase gradients along both radial and azimuthal directions increase, which is significantly different from the common optical vortex beams. Moreover, the HC phase profiles with *K* = 0 and *K* = 1 are distinguished by the phase structure in the central area although both of them have spiral phase structures outside. For *K* = 0, the contour lines tend to repel from each other and there is no central vortex structure, while for *K* = 1, the contour lines join together at the center and there is a vortex structure with TC of *l* presenting at the center. Figure 2a shows the designed phase profile with the parameters of *K* = 1 and *l* = 3, and the SEM images of the fabricated metasurface sample are shown in Fig. 2(c).

### Measurement of 3D twisted intensity profile and complex phase structure

**Figure 3:**
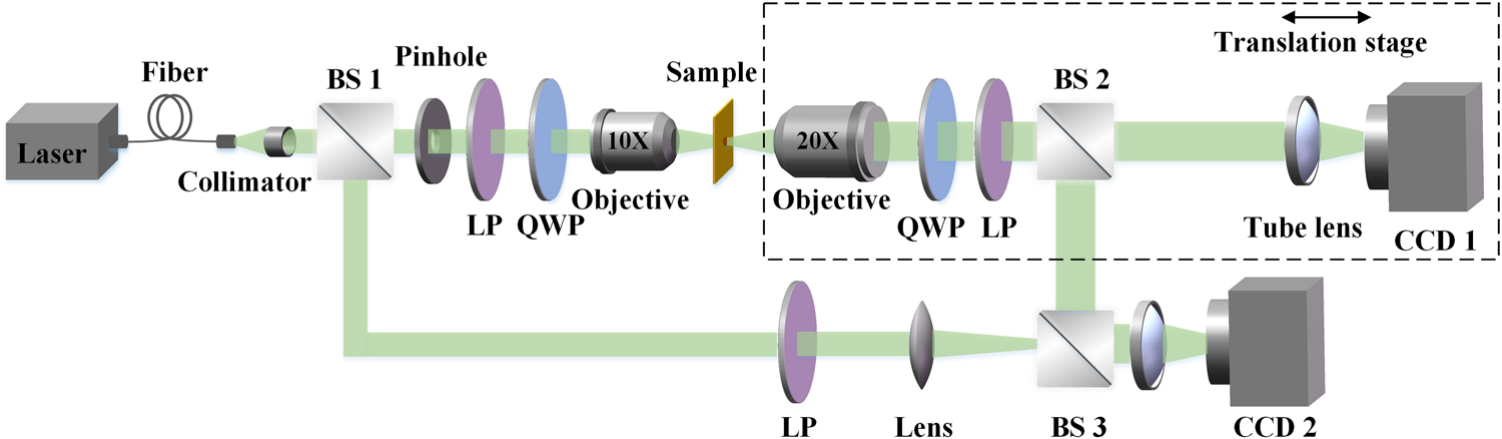
Schematic of the experimental setup.

The 3D twisted structures of the generated HC beams by the metasurfaces are then demonstrated. Figure 3 shows the schematic of experimental setup. The collimated optical beam from a laser diode at the wavelength of 405 nm, 532 nm or 633 nm is first transmitted through a linear polarizer and a quarter-wave plate to create a LCP beam, which is focused onto the metasurface by a 10X objective lens. The generated focused HC beam is obtained by passing through the RCP filter including another set of a quarter-wave plate and a linear polarizer. The 3D twisted beam structure is then observed by a microscope imaging system including a 20X objective lens, a 0.5X tube lens and a CCD camera (CCD 1), which is placed on a translation stage behind the metasurface. The beam intensity profiles at different propagation distances are recorded and assembled for constructing the 3D twisted beam tomography. The interferometry pattern of HC beam is obtained by using the Mach–Zehnder interferometer, where the first beam splitter (BS1) splits the incident beam into the signal beam to generate the HC beam and the reference spherical beam. The second beam splitter (BS2) reflects the generated HC beam to the third beam splitter (BS3) for obtaining the interferometry pattern recorded by another CCD camera (CCD 2).

**Figure 4:**
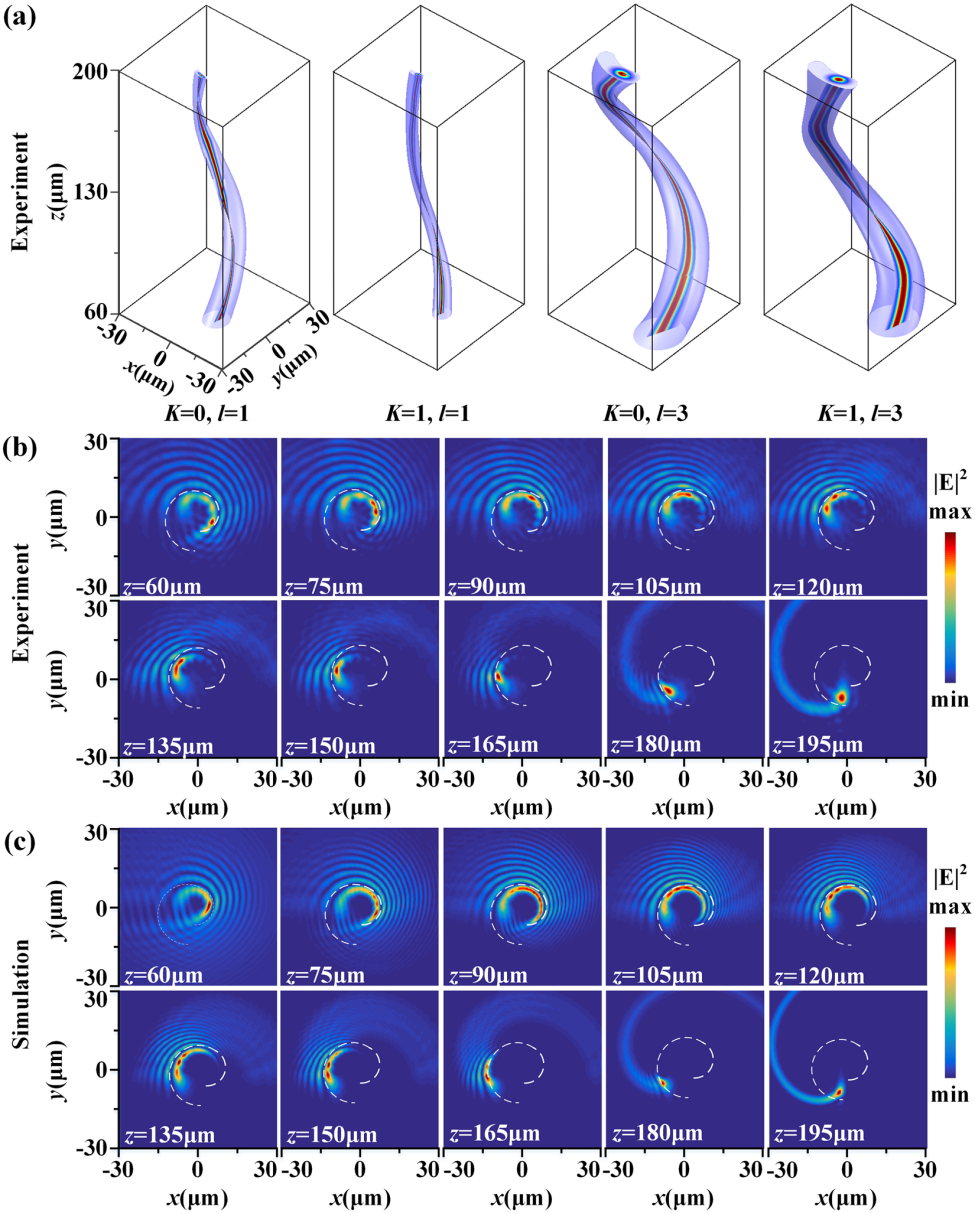
(**a**) From left to right, the 3D twisted tomography for the focused HC beam with parameter set of (*K* = 0, *l* = 1), (*K* = 1, *l* = 1), (*K* = 0, *l* = 3) and (*K* = 1, *l* = 3) at 532 nm. (**b**) Measured and (**c**) simulated cross-sectional intensity profiles for the HC beam with (*K* = 1, l = 3) at different propagation distances *z*.

The measured 3D twisted tomography for the HC beams with four different parameter sets of (*K* = 0, *l* = 1), (*K* = 1, *l* = 1), (*K* = 0, *l* = 3), and (*K* = 1, *l* = 3) at 532 nm is shown in Fig. 4(a), covering the beam propagation distance from *z* = 60 μm to *z* = 200 μm. It is shown that the location of the maximum beam intensity continuously twists around the central propagation axis, and the twisted intensity trajectory is nearly a conical helix which is controlled by the parameters of HC beam. As the TC of HC beam increases from 1 to 3, the size of the twisted intensity structure expands almost three times larger. The parameter *K* only makes the 3D twisted beam shape slightly changed but almost does not affect the size of the twisted structure. Figure 4(b) displays the measured cross-sectional intensity profiles for the HC beam with (*K* = 1, *l* = 3) at different propagation distances *z* at 532 nm, while Fig. 4(c) shows the corresponding calculated results by using the beam diffraction integration method. The dashed white line represents the projection of twisted trajectory onto transverse plane, and it shows a spiral shape. As the beam propagates in the free space, its intensity profile also changes with respect to the propagation distance. At the starting position the intensity profile has many side lobes, then the side lobes are reduced and finally only the main spiral lobe is observed close to the focal plane of *z* = 200 μm.

**Figure 5:**
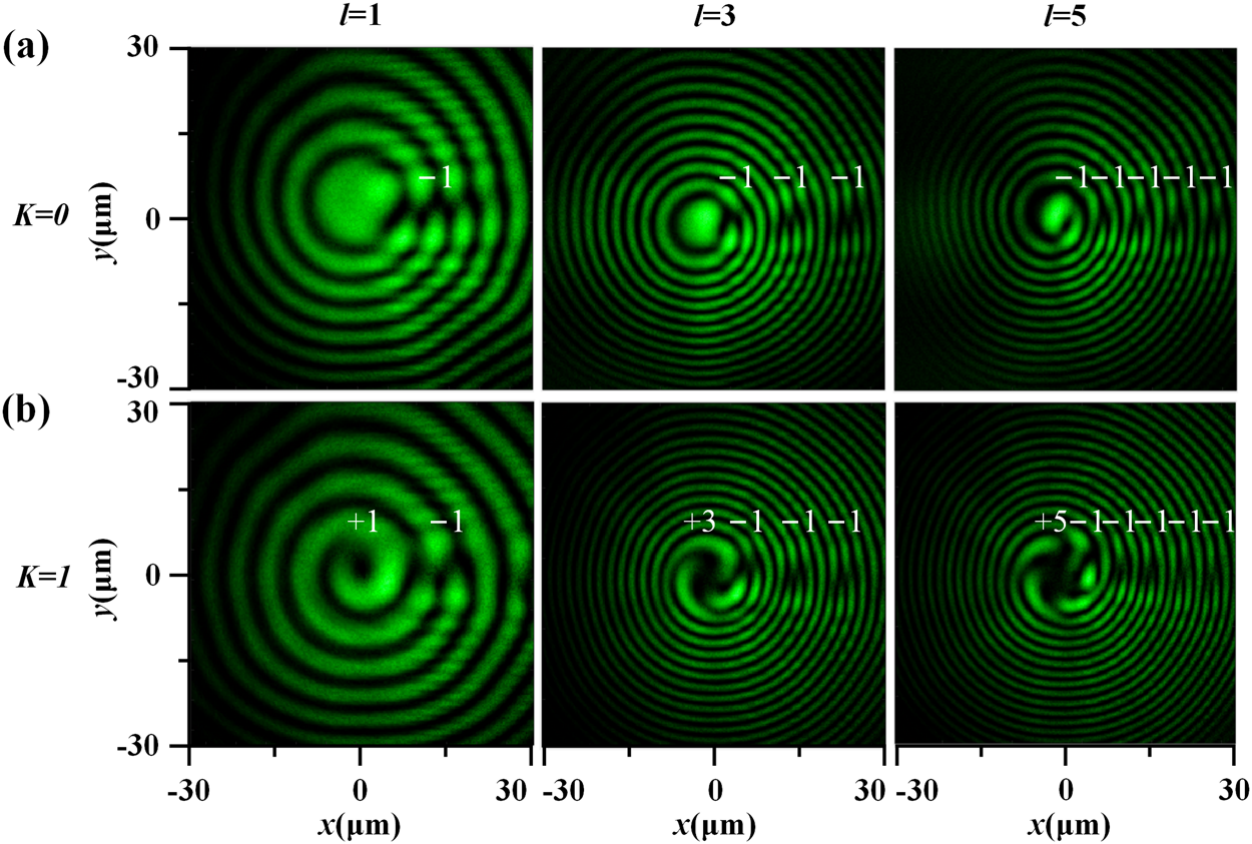
The recorded interferometry patterns of HC beams at *z* = 50 μm for (**a**) *K* = 0 and (**b**) *K* = 1. From left column to right column, *l* changes from 1, 3 to 5.

Next, the generated HC beam is interfered with the reference spherical beam through a Mach–Zehnder interferometer at 532 nm. Figure 5 presents the recorded interferometry patterns at *z* = 50 μm, showing the results of *K* = 0 and *K* = 1 with TC changes as *l* = 1, 3 and 5. For *K* = 0, there is no vortex structure presenting in the beam center, and the peripheral vortex string containing −1 charged single vortices with the total number of *l* is observed along the horizontal phase discontinuous cut line, showing the total OAM of −*lħ*. On the other hand, for *K* = 1, besides the −1 charged peripheral vortex chain, there is one center vortex with charge of +*l* showing up, so that the total number of vortices is *l + *1 and the overall OAM vanishes. For *K* = 0 the peripheral vortices with the same charge repel to each other, while for *K* = 1 the peripheral vortices having the opposite charge as the central vortex will be attracted to the beam center. It is noted that the observed single-charged vortex chain with the same TC located at the phase discontinuous cut line in HC beam is different from that of the fractional vortex beam, where a vortex chain with alternating TC of +1 and −1 is presented in the phase cut line. Another difference is that the number of the vortices in the vortex chain is infinite for the fractional vortex beam^56^, but it is equal to the TC for the HC beam. And the reason for these differences lies in the distinct phase profiles between the HC beam with radially varying phase and the fractional vortex beam with radius-independent phase, although both beams have the phase edge dislocation along radial direction. In addition, the obtained complex phase structures of HC beams are very different from the common optical vortex beam where only single +*l* charged vortex is presented.

**Figure 6:**
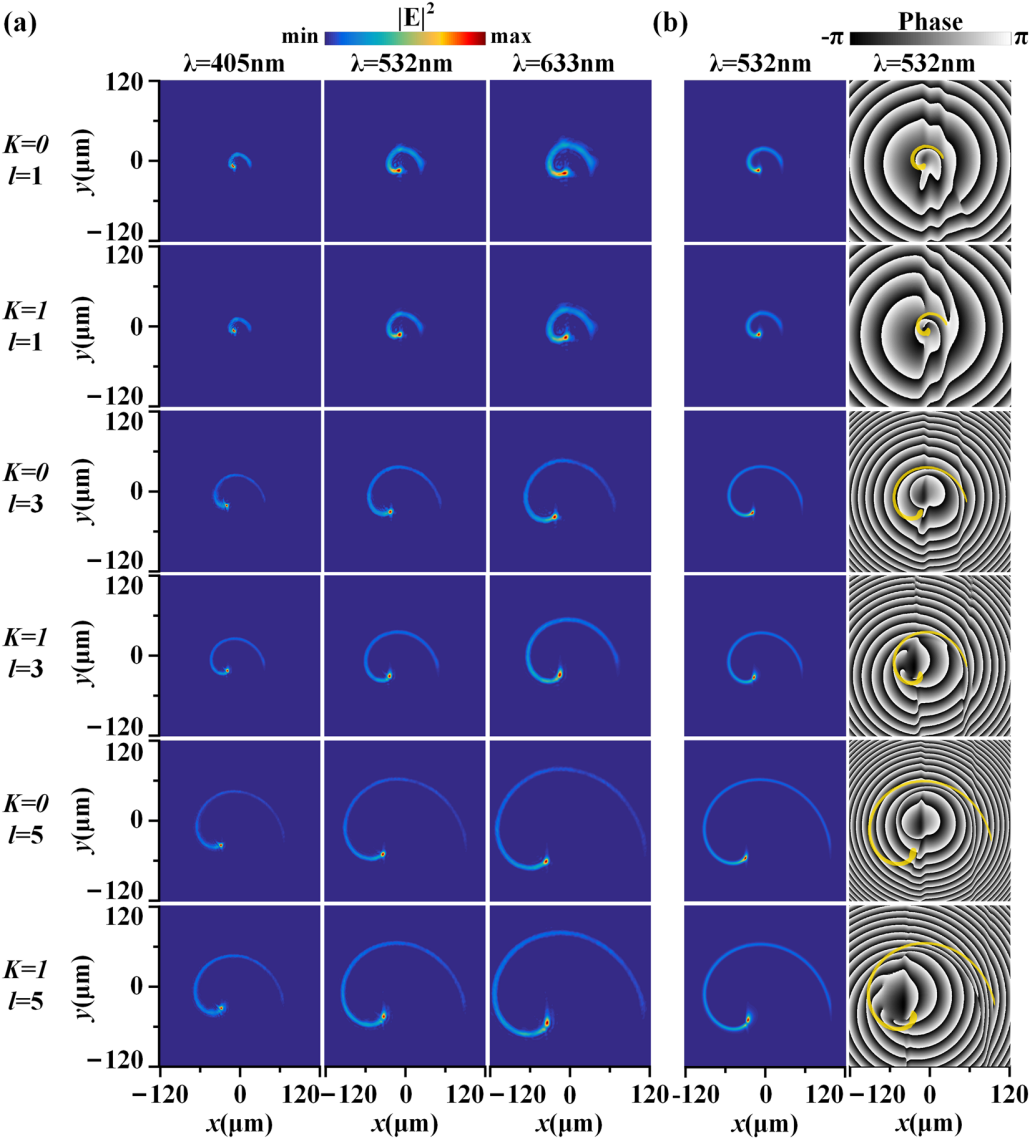
(**a**) Measured intensity profiles at the focal plane of *z* = 200 μm. Form the first row to the last row, the corresponding HC metasurface parameters change with the order: (*K* = 0, l = 1), (*K* = 1, *l* = 1), (*K* = 0, *l* = 3), (*K* = 1, *l* = 3), (*K* = 0, *l* = 5) and (*K* = 1, *l* = 5). And from left column to right column, the laser wavelength changes with the order 405 nm, 532 nm and 633 nm. (**b**) Simulated intensity and phase profiles at the focal plane of *z* = 200 μm at 532 nm. The yellow spiral curves in the phase profiles represent the intensity trajectories.

Finally, the broadband response of the metasurface is demonstrated at three different visible wavelengths of 405 nm, 532 nm and 633 nm. Figure 6(a) shows the measured cross-sectional intensity distributions of HC beams at the focal plane of *z* = 200 μm at three different wavelengths for all six different metasurface samples with parameter sets of (*K* = 0, *l* = 1), (*K* = 1, *l* = 1), (*K* = 0, *l* = 3), (*K* = 1, *l* = 3), (*K* = 0, *l* = 5) and (*K* = 1, *l* = 5). It is observed that the HC beam with varying TC at each wavelength always shows an arithmetic intensity spiral at the focal plane, indicating the broadband operation capabilities of the metasurface. The size of the arithmetic spiral gets expanded as either the TC or the wavelength increases. It is found that with the same TC and wavelength, the parameter *K* does not affect the size of the arithmetic spiral much, and it only changes the intensity profile near the beam center. It is also shown that for the HC beam with any TC, the maximum intensity remains concentrated at the leading point of the arithmetic spiral, which indicates that high photon density keeps localized at a tiny interaction region and will not spread out as the TC increases. Such unique feature of the HC beam is useful for enhanced light-matter interactions where both high photon density and large OAM are necessary. Figure 6(b) plots the simulated intensity and phase profiles at the focal plane of *z* = 200 μm at 532 nm. The phase profiles for both *K* = 0 and *K* = 1 cases indicate that the peripheral vortex strings containing single-charged vortices are not observed in the beam area due to the diffraction at the far field. However, for *K* = 1 case, the original center vortex with charge of +*l* is now decomposed into single-charged vortices with the total number of *l* distributed along the arithmetic intensity spiral, while the vortex singularity centers are located inside of the spiral curve.

## Discussion

In summary, we have demonstrated the direct generation of 3D focused HC beams across the visible wavelength range by using aluminum plasmonic metasurfaces consisting of nanoslit antennas. The designed metasurface is encoded with the combined phase profile by superimposing the HC phase function and a Fourier transform lens. The 3D spiral intensity distributions and complex phase structures of HC beams have been analyzed in detail. The demonstrated HC beam generation with aluminum metasurfaces will find many potential applications in broadband OAM-based light-matter interactions such as particle manipulation and transport, quantum optics and optical communications.

## Methods

### Simulations

The simulations shown in Fig. 1 are conducted by the CST Microwave Studio, where periodic boundary conditions are employed along both *x* and *y* directions in the unit cell. The permittivity of aluminum is taken from experimental data and the refractive index of glass substrate is 1.45. The beam propagation in free space shown in Fig. 4c is calculated by using the Fresnel-Kirchhoff diffraction integral:

4 $$U(x,y,z)=\frac{1}{i\lambda }{\iint }_{S}U({x}_{0},{y}_{0})[\frac{\cos (\overrightarrow{{\bf{n}}}{\boldsymbol{,}}{\bf{r}})-\,\cos (\overrightarrow{{\bf{n}}}{\boldsymbol{,}}{\bf{r}}{\boldsymbol{^{\prime} }})}{2}]\frac{{e}^{ikr}}{r}dS$$

where $U({x}_{0},{y}_{0})$ is the complex amplitude distribution located at the *z* = 0 plane with surface area *S* and normal direction $\overrightarrow{n}$, ${\bf{r}}{\boldsymbol{^{\prime} }}$ is the vector between the source point and a point in the *z* = 0 plane, ${\bf{r}}$ is the vector between the point in *z* = 0 plane and a point in the plane at the propagation distance *z*, and *k* = 2π/*λ* is the wavevector.

### Sample fabrication

The metasurface is made in a 35 nm-thick aluminum film deposited on a glass substrate using electron-beam evaporation. The nanoslit arrays are milled in the aluminum film using focused ion beam (FIB) system (FEI Helios Nanolab 600, 30 kV, 93 pA). The metasurface contains 208 × 208 unit cells, and each unit cell contains a milled nanoslit with size of 160 nm × 8 nm at a specified orientation angle.

### Optical characterization

The transmission spectra through the metasurface under circular polarization basis in Fig. 1 are measured with a collimated broadband Tungsten-Halogen source, where a combination of a linear polarizer and an achromatic quarter-wave plate are used to convert the incident light to circularly polarized wave. The light beam is focused normally onto the sample using a 50× objective lens and the transmitted light is collected by another 10× objective lens to a spectrometer (Horiba, iHR 550). Another set of a quarter-wave plate and a linear polarizer is used to distinguish the original and converted circular polarization components. A transparent glass substrate is utilized to normalize the transmission spectra. Since the metasurface operates in a broad wavelength range from 400 nm to 800 nm, three lasers operating at different wavelengths of 405 nm, 532 nm and 633 nm are employed in the HC beam experiments, as shown in Fig. 3. The 3D twisted beam structures are captured by a microscope imaging system with a 20X objective lens, a 0.5X tube lens and CCD camera placed on a translation stage. The interferometry pattern of HC beam is obtained by using the Mach–Zehnder interferometer.
